# Respiratory syncytial virus prophylaxis for prevention of recurrent childhood wheeze and asthma: a systematic review

**DOI:** 10.1186/s13643-020-01527-y

**Published:** 2020-11-25

**Authors:** Lauren Alexandra Quinn, Michael D. Shields, Ian Sinha, Helen E. Groves

**Affiliations:** 1 Queen’s University Belfast, University Road, BT71NN Belfast, Northern Ireland; 2 Centre for Experimental Medicine, Welcome Wolfson Institute for Experimental Medicine, Queen’s University Belfast, 17 Lisburn Road, Belfast, BT97BL Northern Ireland; 3 University of Liverpool, Liverpool, England

**Keywords:** Respiratory syncytial virus, asthma, Recurrent wheeze, Prophylaxis, Monoclonal antibody, Immunoprophylaxis

## Abstract

**Background:**

Acute bronchiolitis caused by respiratory syncytial virus (RSV) has been associated with greater risk of recurrent wheezing and asthma. However, it is unclear whether this association is causal. RSV-specific monoclonal antibodies have been shown to reduce RSV-related hospitalisations in high-risk infants, but the longer-term follow-up has given conflicting evidence for prevention of recurrent wheeze or asthma.

**Objective:**

We performed a systematic review and meta-analysis to determine whether monoclonal antibody prophylaxis against RSV bronchiolitis reduces the risk of subsequent recurrent wheeze or asthma. If so, this may support the hypothesis of causality.

**Methods:**

Studies were identified via an online database search using Embase, MEDLINE, PubMed, Web of Science and the Cochrane Library. Manufacturers of monoclonal antibodies were contacted directly for unpublished data. The intervention of interest was RSV monoclonal antibody prophylaxis, and the primary outcome measure was recurrent wheeze and/or asthma. Studies were screened according to inclusion/exclusion criteria. Included studies were evaluated for quality and assessed for bias independently by 3 reviewers using the ‘Grading of Recommendations Assessment, Development and Evaluation’ (GRADE) approach. Results were extracted into 2 × 2 outcome tables and a meta-analysis carried out producing forest plots based on relative risk. Heterogeneity was assessed using the *I*^2^ statistic.

**Results:**

The search identified 141 articles, which, after screening, resulted in eight studies (2 randomised controlled trials), thus including 11,195 infants in the meta-analysis. The overall result demonstrated a non-statistically significant reduction in relative risk of developing recurrent wheeze or asthma (RR 0.60; 95% CI 0.31 to 1.16). Study quality was generally low with evidence of publication bias and statistical heterogeneity. However, sub-group analysis excluding studies deemed to be ‘very low’ quality showed a relative risk of 0.42 (95% CI 0.22 to 0.80, *p* = 0.008). A further sub-group analysis for infants aged 32 to < 36 weeks showed a statistically significant relative risk of 0.35 (95% CI 0.14 to 0.86, *p* = 0.02).

**Discussion:**

We did not identify an overall statistically significant benefit. However, our two sub-group analyses did find statistically significant benefits of monoclonal antibody therapy on the risk of recurrent wheeze and asthma. The main limitation of this study is the lack of high-quality randomised controlled trials, highlighting the need for more research in this field.

**Supplementary Information:**

The online version contains supplementary material available at 10.1186/s13643-020-01527-y.

## Background

Bronchiolitis, an acute lower respiratory tract infection (LRTI), is the most common reason for hospitalisation in young children in many countries including the UK and USA [1]. The majority of young children will experience bronchiolitis, with approximately 3% requiring hospital admission [1]. LRTIs in early life, particularly in infancy, are associated with development of recurrent wheeze and asthma in later childhood [2]. Pre-term infants especially are at an increased risk of both severe bronchiolitis and recurrent wheeze or asthma development independently [3, 4].

Recurrent wheeze in infancy is common, with one large international study of infants reporting that 45% had at least one wheezing episode, and 20% had three or more [5]. This can have a substantial effect on health-related quality of life for infants and their families [6]. When recurrent attacks of wheezing continue to occur as the child ages, an asthma diagnosis becomes more likely. Asthma is the most prevalent chronic respiratory disease worldwide, and its pathogenesis is multifactorial with hypersensitivity and inflammation of the airways leading to wheeze and shortness of breath [7, 8]. It has been estimated that the cost of asthma is approximately £1.1 billion in the UK, highlighting it as a key public health issue [9]. It creates a huge burden on both patients and health services in terms of health-related quality of life and cost, with the most significant impact being amongst lower socioeconomic groups and ethnic minorities [10]. With the overall prevalence increasing globally, further research is needed into why this increase is happening, and whether or not there any preventative measures that can be undertaken [10].

Acute bronchiolitis in early life is very strongly associated with an increased risk of asthma development [11]. It has been shown that infants hospitalised with acute bronchiolitis have a significantly increased risk of developing childhood wheeze and asthma, with one study from Finland finding the risk of recurrent wheeze or asthma development post hospitalised bronchiolitis to be twice that of the general population [12]. However, while this association is well established, debate remains over whether acute bronchiolitis is merely the first manifestation of recurrent wheeze or asthma or contributes to causation. To assess causality, studies assessing the impact of bronchiolitis prevention on the outcome of subsequent development of recurrent wheeze and asthma are needed [11].

The most common cause of acute bronchiolitis is respiratory syncytial virus (RSV), primarily occurring in infants up to 12 months. RSV-specific monoclonal antibodies provide passive immunity and have shown efficacy in reducing RSV hospitalisations in high-risk infants, such as those born prematurely [13–15]. Currently, immunoprophylaxis is only deemed cost-effective in very early pre-term infants (< 32 weeks’ gestational age) for prevention of severe RSV bronchiolitis. Palivizumab is the most common RSV-specific monoclonal antibody in use and has been shown to be well tolerated with very low rates of minor adverse events such as injection site reaction, fever, diarrhoea, and irritability [16]. Motavizumab, a palivizumab derivative, is a second-generation humanised monoclonal antibody. Initially hoped to have greater efficacy and lower dose requirement when compared to palivizumab [16, 17], motavizumab was discontinued in 2010 due side effect concerns, particularly in regard to serious skin reactions, and doubts over superior efficacy to palivizumab [16, 18, 19]. Other palivizumab biosimilars have been developed including nirsevimab, which has a longer half-life than palivizumab [20]. Lunamab is another RSV-specific monoclonal antibody developed as a less-expensive biosimilar aimed at low-income countries [21]. Another biosimilar antibody, suptavumab, was withdrawn in 2017 due to failure to meet primary endpoint in clinical trial testing [22].

Monoclonal antibodies are prohibitively expensive and the estimated cost of palivizumab is approximately £3000–£5000 ($3700–$6200 USD) per child [23]. Despite proven efficacy and the high prevalence of RSV infection in infancy, most children will not experience a severe RSV-related illness and thus delivery of monoclonal antibody therapy to all infants is not currently considered cost-effective [23, 24]. Smart et al. in their systematic review of the cost-effectiveness of RSV prophylaxis on the outcome of RSV bronchiolitis found it to be cost-effective for high-risk groups including very early pre-term infants (< 32 weeks), children with congenital heart disease and those of Aboriginal descent [25]. They also noted for infants born at 33–35 weeks gestational age, RSV prophylaxis could be considered cost-effective in the presence of additional risk factors including chronological age, number of siblings, history of atopy, absence of breast feeding, cigarette smoke exposure and daycare attendance [26].

To further examine the relationship between RSV bronchiolitis and subsequent development of recurrent wheeze and asthma, we conducted a systematic review and meta-analysis to determine whether monoclonal antibody RSV prophylaxis compared with no prophylaxis, in infants born from early pre-term up to term, reduces the risk of recurrent wheeze or asthma development in later childhood.

## Methods

### Protocol and registration

The review protocol is registered with PROSPERO (CRD42019i28239) and is accessible at https://systematicreviewsjournal.biomedcentral.com/articles/10.1186/s13643-019-1251-x. Findings are reported according to the Preferred Reporting Items for Systematic Review and Meta-Analysis (PRISMA) standards (Additional file 1).

### Inclusion criteria

#### Types of studies and participants

Table 1 summarises the inclusion/exclusion criteria used in study screening. Primary studies including randomised control trials (RCTs), prospective observational case-control studies and cohort studies were included. We only included studies enrolling pre-term infants (up to full term), who were subsequently followed up until infancy or childhood (1–10 years).

**Table 1:** Inclusion and exclusion criteria used when screening articles first by title and abstract and then by full text. Papers were included if they were primary studies of any study design. The population being studied was infants born early pre-term up to term. The studies were investigating monoclonal antibody prophylaxis compared with no prophylaxis or placebo, on the outcome of recurrent wheeze or asthma. No studies investigating a population of infants with congenital defects were included, and no other RSV prophylaxis or treatment apart from monoclonal antibody was considered

Include	Exclude
All study designs	Reviews
Primary studies, including peer-reviewed and grey literature	Letters
All ethnicities	Not about prophylaxis
Population: Infants born early pre-term up to term, followed up for 1–10 years	Population: Infants with congenital heart defects or lung conditions such as bronchopulmonary dysplasia
Intervention: RSV prophylaxis with monoclonal antibody	Any other interventions such as RSV Prophylaxis or treatment with RSV-specific immune globulins, steroids, vaccines, macrolides etc.
Comparison: No prophylaxis or placebo	Comparison: Different dosing regimen of monoclonal antibody
Outcome: Recurrent wheeze or asthma development	Bronchiolitis caused by other allergens or viruses such as rhinovirus

#### Intervention, comparison and outcome

The intervention being investigated was the use of RSV-specific monoclonal antibodies for RSV immunoprophylaxis compared to no RSV prophylaxis or placebo. The only outcome measured was development of subsequent recurrent wheeze and/or asthma as defined by the study authors, including parent-reported wheeze as well as formally doctor-diagnosed wheeze or asthma. Parent-reported wheeze is an important outcome to include as not all infants who wheeze will be assessed by a physician. Other outcomes such as RSV hospitalisation or allergy diagnosis were assessed in some studies; however, these are not a priority for this review.

### Information sources and search strategy

The literature search, using the strategy in Table 2, was conducted using Embase, MEDLINE, PubMed, Web of Science and the Cochrane Library. We also contacted the manufacturers of RSV-specific monoclonal antibodies for any unpublished data, and searched trial registries such as ‘ClinicalTrials.gov’ and ‘BMC Trials’ and the WHO International Clinical Trials Registry, for any potentially suitable studies that may be imminently reported and published. The date last searched was July 2020. Reference lists of included and other relevant papers were hand searched to identify possible additional primary studies.

**Table 2:** Search strategy. An example of the comprehensive literature search performed across the electronic databases, with search terms and limitations applied. Shown search strategy example is from MEDLINE

#	Searches	Results
1	Respiratory Syncytial Virus Infections/	6766
2	"RSV infection".mp. [mp = title, abstract, original title, name of substance word, subject heading word, floating sub-heading word, keyword heading word, organism supplementary concept word, protocol supplementary concept word, rare disease supplementary concept word, unique identifier, synonyms]	3688
3	Asthma/	123943
4	"asthma development".mp. [mp = title, abstract, original title, name of substance word, subject heading word, floating sub-heading word, keyword heading word, organism supplementary concept word, protocol supplementary concept word, rare disease supplementary concept word, unique identifier, synonyms]	748
5	wheeze.mp. [mp = title, abstract, original title, name of substance word, subject heading word, floating sub-heading word, keyword heading word, organism supplementary concept word, protocol supplementary concept word, rare disease supplementary concept word, unique identifier, synonyms]	13574
6	Respiratory Hypersensitivity/	9559
7	atopy.mp. [mp = title, abstract, original title, name of substance word, subject heading word, floating sub-heading word, keyword heading word, organism supplementary concept word, protocol supplementary concept word, rare disease supplementary concept word, unique identifier, synonyms]	10555
8	1 or 2	7938
9	3 or 4 or 5 or 6 or 7	144279
10	8 and 9	948
11	limit 10 to english language	879
12	limit 11 to "all child (0 to 18 years)"	600
13	limit 12 to journal article	558
14	(later or subsequent).mp. [mp = title, abstract, original title, name of substance word, subject heading word, floating sub-heading word, keyword heading word, organism supplementary concept word, protocol supplementary concept word, rare disease supplementary concept word, unique identifier, synonyms]	1217789
15	risk factors/	811027
16	"clinical factor".mp. [mp = title, abstract, original title, name of substance word, subject heading word, floating sub-heading word, keyword heading word, organism supplementary concept word, protocol supplementary concept word, rare disease supplementary concept word, unique identifier, synonyms]	16674
17	14 or 15 or 16	1992676
18	13 and 17	255
19	prophylaxis.mp.	106030
20	Primary Prevention/	18238
21	monoclonal antibody.mp. or Antibodies, Monoclonal/	235160
22	palivizumab.mp. or Palivizumab/	1066
23	motavizumab.mp.	59
24	prevention.mp.	1619597
25	19 or 20 or 24	1664918
26	21 or 22 or 23	235631
27	25 and 26	12613
28	18 and 27	31

### Data collection and analysis

#### Selection of studies

Studies were independently screened according to inclusion and exclusion criteria by 2 independent reviewers (HG and MS). The screening was a 2-step process, first by title and abstract, and then by full text. A third-party reviewer was involved in the case of any disagreements. Duplicate articles, identified using reference software, were removed.

#### Data extraction and management

Data were extracted using an adapted form of the ‘Data collection form for Intervention review—RCTs and non-RCTs’ of the Cochrane Collaboration [27] (Additional file 2). Data is presented in the summary of findings table which includes study type, population number, number in intervention and comparison groups, 2 × 2 outcome tables, relative risk and evaluation of the quality of evidence and bias risk as per Cochrane handbook guidance [28].

#### Risk of bias

Study validity was evaluated using the Cochrane Risk of Bias table, and overall quality of evidence was evaluated independently by 3 reviewers using the ‘Grading of Recommendations Assessment, Development and Evaluation’ (GRADE) approach as detailed in our protocol [29].

#### Data synthesis and meta-analysis

Using the main outcome of recurrent wheeze (dichotomous—yes/no) and the data from the 2 × 2 outcome tables produced, a meta-analysis was performed using a random effects model, with relative risk as the principal summary measure. Individual studies are represented on a forest plot based on relative risk and 95% confidence intervals. Funnel plots were generated to portray publication bias or possible selective reporting within studies. StatsDirect statistical software was used for the meta-analysis [30].

#### Heterogeneity

To test for heterogeneity (inconsistency between studies), we used the *I*^2^ test, taking an *I*^2^ of > 75% as being high heterogeneity.

#### Sub-group analysis

Sub-group analysis of late pre-term infants was performed to explore the effectiveness of RSV prophylaxis on subsequent recurrent wheeze in this population. Sub-group analysis excluding very low-quality evidence was also carried out, due to the risk of confounding by indication.

## Results

**Fig. 1:**
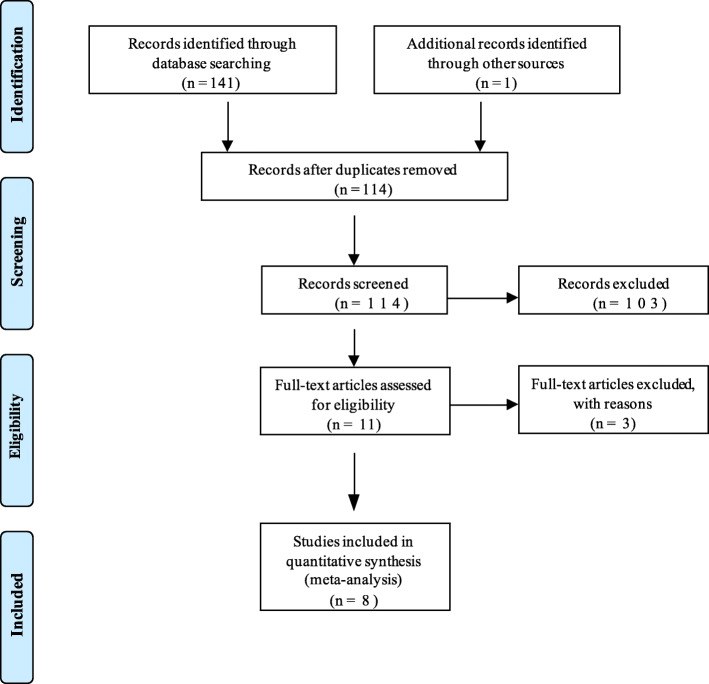
PRISMA flow diagram. Study screening and selection outlined using the Preferred Reporting Items for Systematic Reviews and Meta-Analyses (PRISMA) flow diagram [31]. This process resulted in 8 studies being included in the final quantitative meta-analysis

The search across the main databases generated 141 references. After removal of duplicate papers and full-text screening, eight studies were included in this review (Fig. 1). These included two RCTs, four cohort studies, one case-control study and one cross-sectional study. Reasons for the studies excluded after full-text review are outlined in Additional file 2. Study characteristics for which data was extracted are presented in the summary of findings table (Table 3).

**Table 3:** Summary of findings table. Study characteristics for which data was extracted for each study are presented in this table. These include number of participants, length of follow-up, outcomes, relative risk and the GRADE assessment for quality of evidence and bias risk

RSV prophylaxis for prevention of recurrent childhood wheezing								
Population: Pre-term and term infants Intervention: RSV prophylaxis (palivizumab/motavizumab) Comparison: No RSV prophylaxis								
Study	No. of participants	Follow-up period	Outcomes	Intervention	Control	Relative risk (95% CI)	Quality of evidence GRADE	Comments
Simoes EAF [32]	421	2 Years	*Wheeze*	25/191	59/230	0.51 (CI = 0.33 to 0.78)	LOW ++	Observational cohort study
			*No wheeze*	166/191	171/230			
O’Brien KL [33]	1919	3 Years	*Wheeze*	35/1278	16/641	1.10 (CI = 0.61 to 1.97)	HIGH ++++	Randomised control trial
			*No wheeze*	1243/1278	625/641			
Mochizuki H [34]	440	6 Years	*Wheeze*	44/345	68/95	0.18 (CI = 0.13 to 0.24)	LOW ++	Case control
			*No wheeze*	301/345	27/95			
Carroll KN [35]	6566	6 Years	*Wheeze*	1056/4222	441/2344	1.33 (CI = 1.20 to 1.47)	VERY LOW +	Cohort study • Confounding by indication
			*No wheeze*	2966/4222	1902/2344			
Scheltema NM [36]	395	6 Years	*Wheeze*	28/199	47/196	0.59 (CI = 0.38 to 0.90)	HIGH ++++	Randomised control trial
			*No wheeze*	171/199	149/196			
Igde M [37]	339	3 years	*Wheeze*	2/113	26/226	0.15 (CI = 0.038 to 0.63)	LOW ++	Observational study
			*No wheeze*	111/113	200/226			
Simoes MC [38]	445	3 Years (average)	*Wheeze*	70/194	52/251	1.74 (CI = 1.28 to 2.37)	VERY LOW	Observational cross-sectional study • Confounding by indication
			*No wheeze*	124/194	199/251			
Moreno-Galdo A [39]	670	3 Years	*Wheeze*	7/108	82/562	0.44 (CI = 0.21 to 0.90)	LOW	Observational study
			*No wheeze*	101/108	480/5562			

In total, 11,195 infants were included in this review. The gestational age of participants ranged from 24 weeks to full term and the median length of follow-up across the studies was 4 years (range 2 to 6 years). Of the 8 studies, 5 reported a reduction in recurrent wheeze after RSV-specific monoclonal antibody prophylaxis.

**Fig. 2:**
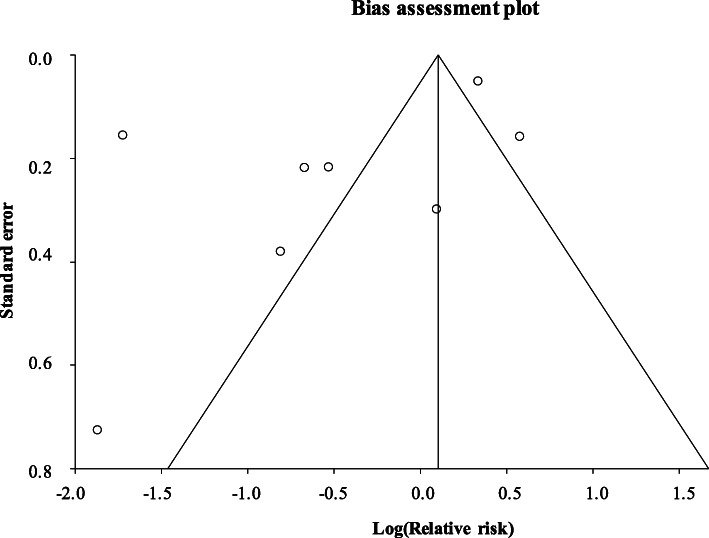
Publication bias. Funnel plot highlighting the risk of publication bias in results. More precise results are plotted near the top (lower standard error). Points plotted outside the funnel indicate high risk of publication bias

Quality of evidence was variable. The two RCTs were considered as high-quality evidence, with most other studies graded as low-quality evidence. The funnel plot in Fig. 2 is likely to reflect publication bias, but could also represent selective reporting within studies, or poor methodology, which is in keeping with the GRADE assessment of bias outlined in Table 3. We noted a significant number of manufacturer-funded studies creating the potential for sponsorship bias. There was considerable statistical heterogeneity between studies (*I*^2^ 96.6%; 95% confidence interval [CI] 95.4 to 97.3%).

**Fig. 3:**
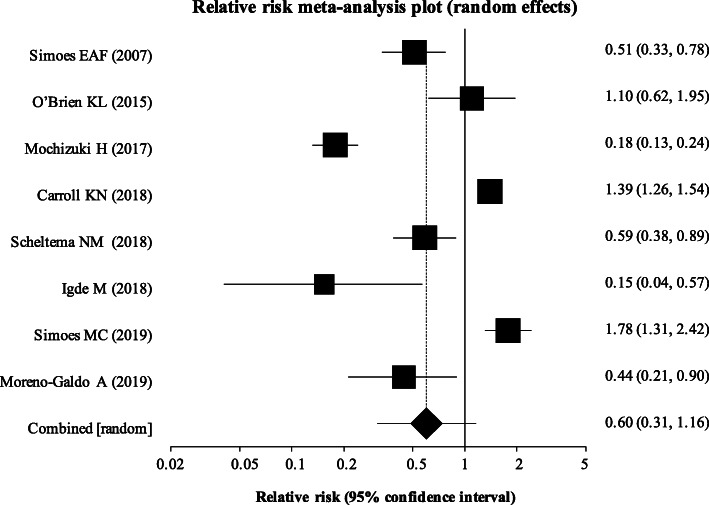
Overall result of meta-analysis. Using a random effects model, a meta-analysis was performed and a forest plot constructed using relative risk as the summary measure. This shows a pooled relative risk for the primary outcome of 0.60 with use of monoclonal antibody prophylaxis; however, results are not statistically significant

Figure 3 displays the forest plot of pooled relative risks, derived using a random effects model to allow for heterogeneity. Overall, RSV monoclonal antibody prophylaxis did not confer a statistically significant benefit on the reduction of the risk of recurrent wheeze or asthma (relative risk [RR] 0.60; 95% CI 0.31 to 1.16, *p* = 0.129).

### Sub-group analyses

**Fig. 4:**
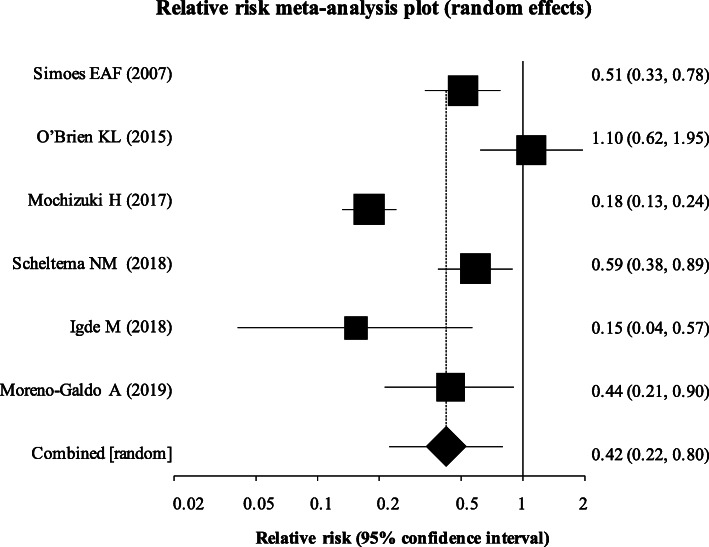
Meta-analysis with very low-quality studies removed. Two studies carried a significant risk of bias by confounding, skewing the results and showing the opposite effect to the overall result. Upon removal of these studies in a sub-group analysis, the overall pooled relative risk became statistically significant at 0.42 (95% CI = 0.22 to 0.80)

Two studies (Carroll et al. and Simoes et al.) [35, 38] were graded as being of very low quality with serious risk of bias. Carrol et al.’s study was deemed to show considerable risk of bias given infants most at risk of both RSV and development of recurrent wheeze were more likely to have increased uptake and compliance with monoclonal antibody prophylaxis, potentially skewing results. In the report by Simoes et al., there was a serious risk of confounding due to the fact that the average gestational age of the palivizumab-treated group was 28 weeks, compared with 34 weeks in the untreated group such that infants in the treated group were already at an increased risk of RSV and recurrent wheeze. In view of the significant confounding for these two studies, a sub-group analysis excluding these studies was performed (Fig. 4). This resulted in a significant reduction in relative risk of 0.42 (95% CI 0.22 to 0.80) for the primary outcome (*p* = 0.008).

**Fig. 5:**
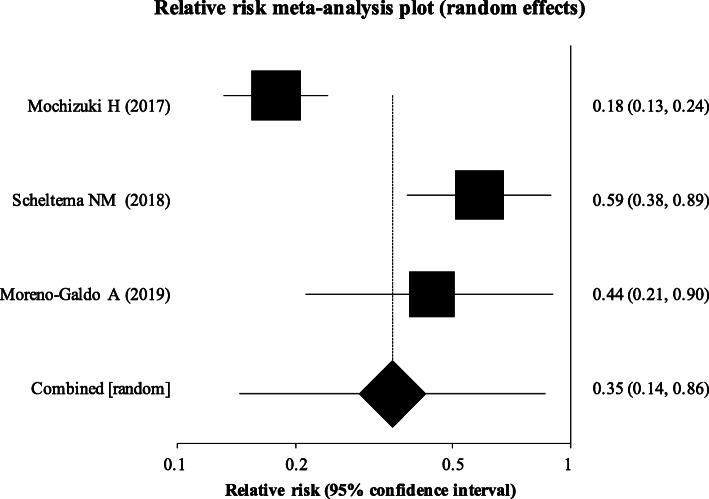
Sub-group analysis among pre-term infants with gestational age 32 to < 36 weeks. This sub-group analysis demonstrates a relative risk of 0.35 (95% CI = 0.14–0.86), showing a statistically significant reduction in risk of recurrent wheeze among this cohort of preterm infants with palivizumab use

To investigate the moderate to late pre-term cohort, a pre-specified sub-group analysis was planned for infants whose gestational age was 33–35 weeks. However, only 3 papers specifically focused on this later pre-term cohort. To allow for this sub-group meta-analysis to be performed using available study data, we adjusted the gestational age range included to be 32 to < 36 weeks. In this sub-group, we found that infants who received monoclonal antibody prophylaxis had a significant reduction in relative risk of developing wheeze or asthma (RR 0.35; 95% CI 0.14 to 0.86, *p* = 0.02) (Fig. 5).

### Safety and adverse effects

Safety data regarding monoclonal antibody use were not reported for most studies, with the exception of one RCT, comparing motavizumab with placebo in 1919 infants, reported by O’Brien et al. In this study, eight serious adverse events were associated with use of motavizumab, all within one day of dosing. Six were consistent with hypersensitivity type reactions, one appeared as erythema multiforme and one was self-resolving skin erythema [33].

## Discussion

This systematic review aimed to evaluate evidence around whether RSV prophylaxis, using monoclonal antibodies, reduced the risk of pre-term infants developing recurrent wheeze or asthma in childhood. According to our primary analysis, including all studies regardless of quality, and infants of all gestational ages, we did not identify a statistically significant benefit for this outcome. However, our two pre-defined sub-group analyses, in which we excluded studies of very low quality, and separately focussed on infants born after 32 weeks gestation, did find statistically significant benefits on monoclonal antibody therapy on the risk of recurrent wheeze and asthma.

We noted three studies whose relative risk results showed a different effect to the overall result. O’Brien et al. showed a relative risk of 1.10 for the primary outcome with monoclonal antibody prophylaxis using motavizumab [33]. They concluded that prophylaxis with motavizumab had no effect on subsequent recurrent wheeze. However, the reported confidence intervals are extremely wide, meaning that the true effect could lie anywhere between 0.61 and 1.97. When assessing quality of this evidence using the GRADE approach, it was concluded as being a high-quality study. It was a RCT with a low risk of recall or sponsorship bias. However, it is important to note it was performed in a population of Native American infants only, who have been shown to be at an increased risk of serious RSV bronchiolitis, with the RSV-associated hospital admission rate being almost 2.5 times that of infants in the general US population of the same age [40, 41]. Another important factor to consider is that this study was the only study investigating motavizumab as the monoclonal antibody prophylaxis. While motavizumab has been proven to be efficacious in terms of reducing RSV hospitalisations in various studies, this study was the first to assess its efficacy in reducing recurrent wheeze [18, 33]. Thus, results for this study may not be as generalizable to use of other monoclonal antibodies in a non-native American population.

Carroll et al. report a cohort study which also appeared to show the opposite effect to the overall pooled effect [35]. When using the GRADE approach to assess the quality of each study, this study was found to have a considerable risk of confounding. The authors report measuring adherence to palivizumab as 0% adherence, < 70% adherence and > 70% adherence; however, due to the specificities of the research question in our systematic review, the < 70% adherence group and the > 70% adherence group were combined. Accordingly, many included patients in the treatment group may not have received the intervention. Furthermore, more infants in the > 70% adherence group had chronic lung disease, lower median birth weight and longer median birth hospital stay and also were generally smaller for gestational age than in the other groups [35]. These are all known risk factors for increased severity of RSV infection and subsequent childhood wheeze [42–45]. Accordingly, this may have skewed results and may account for the observation in this study that palivizumab use was significantly associated with increased risk of recurrent wheeze [35].

The cross-sectional study carried out by Simoes et al. in 2019 also demonstrated a seemingly opposite result to the overall pooled result [38]. When assessing quality of evidence, this paper was also deemed to be very low quality due to confounding in relation to gestational age. There was a significant difference in mean gestational age between the palivizumab-treated group and the untreated group, 28 weeks and 34 weeks respectively. As a result, this study also appears to show that palivizumab use is associated with an increased risk of recurrent wheeze [38].

The remaining studies each showed a significant reduction in relative risk for the primary outcome with use of monoclonal antibody prophylaxis. However, it is important to highlight that the quality of this evidence varied widely and was mostly graded as low-quality evidence, with only one high-quality RCT—the MAKI Trial [36].

Recurrent wheeze represents a huge public and global health problem. It has been estimated that the cost of asthma is approximately £1.1 billion in the UK [9]. On the level of the individual child affected, recurrent childhood wheeze can significantly impact quality of life. It can lead to inability to partake in physical exercise and play and, thus, affect the child’s education and development [46]. There is also substantial burden on the family in terms of working days lost for parents/carers. A recent epidemiological study using electronic health care records to estimate the prevalence of recurrent wheeze found that parent-reported wheezing prevalence was 12.9% [47]. Thus, any intervention to reduce recurrent wheeze prevalence would have the potential to significantly improve the lives of many children and families as well as having considerable financial benefits.

Monoclonal antibodies are expensive, and analyses based on RSV bronchiolitis outcomes suggest their use is only cost-effective for certain high-risk groups, such as early pre-term infants (< 32 weeks). However, later pre-term infants aged between 32 and 36 weeks gestation also have high RSV hospitalisation rates (3.75% and 9.8%) and immunological differences in lung development [48]. Our pre-specified sub-group analysis of 3 studies focusing on infants with gestational age ranging from 32 weeks to 36 weeks showed a statistically significant relative risk of 0.35, suggesting that some infants in this cohort may benefit from RSV prophylaxis to reduce their risk of subsequent recurrent wheeze. Cost-effectiveness analysis of RSV prophylaxis, based on the composite outcome of RSV bronchiolitis and recurrent wheeze in this specific gestational category, is necessary to draw more robust conclusions about this issue.

While there is an established association between RSV infection and subsequent recurrent wheeze, there is question of whether or not this recurrent wheeze can be called asthma. The MAKI trial (Scheltema et al.) was initially published in 2013 as a 1-year follow-up study; however, in this systematic review, we included the 6-year follow-up data. The original 1-year study found a statistically significant reduction in the proportion of infants with recurrent wheeze in the palivizumab-treated group [49]. In the 6-year follow-up study, while a significant difference between treatment and control groups in parent-reported asthma at age 6 years was noted, no significant difference in lung function tests or physician diagnosed asthma was evident [36]. Prais et al. reported in 2016 the results of a study on the short- and long-term effects of palivizumab use in premature infants and reported a significant reduction in rates of recurrent wheeze during the first 2 years of life in those who received palivizumab (27% compared to 70% in the untreated group) [50]. However, by school age (7–10 years), there was no significant difference in recurrent wheeze or pulmonary function tests. This suggests that monoclonal antibody prophylaxis may have a protective effect on the airway short term but not long term. Data from this study were not included in this meta-analysis due to the fact that the majority of infants included were diagnosed with bronchopulmonary dysplasia [50].

RSV infection itself causes direct damage to the lungs, particularly in premature infants, for example ciliary destruction, necrosis of epithelial cells, inflammation of the submucosa and bronchiolar plugging from mucous secretions [50]. Considering this and the fact that studies have shown no significant effect of RSV prophylaxis on lung function at school age, it could be hypothesised that post-RSV recurrent wheeze may not represent atopic asthma but rather results from direct damage of the RSV infection to the lungs which gradually improves with time and age. This is supported by previous work by Martinez et al. who investigated factors affecting wheezing before the age of 3 years and at 6 years and concluded most infants who wheeze do not have an increased risk of asthma or allergy later in life [51]. Using the same study population as Simoes et al. [32], the same research team explored the protective effect of palivizumab prophylaxis on recurrent wheeze in atopic vs non-atopic children. They found that immunoprophylaxis reduced the relative risk by 80% in non-atopic children, but had no effect in those with a family history of atopy [52]. Taking all this into consideration, it may be that RSV prophylaxis with monoclonal antibody reduces the risk of RSV infection in certain groups by preventing the airway from direct damage by RSV and reducing subsequent risk of recurrent wheeze. However, if the mechanism by which RSV causes recurrent wheeze is independent of atopy, in children with an atopic predisposition, RSV prophylaxis would not reduce the risk of subsequent or asthma.

### Study limitations

Firm conclusions from this systematic review are hindered by the lack of good-quality evidence. Only eight studies were eligible to be included in the review and of these 8, only 2 were considered to be of high quality. The significant statistical heterogeneity between the studies and publication bias found is likely to affect the overall pooled results of this meta-analysis, introducing significant risk of bias. Ideally, more RCTs are needed to investigate the role of monoclonal antibody prophylaxis on the outcome of recurrent wheeze. However, such RCTs in at-risk populations would be difficult given the proven benefit of palivizumab in prevention of severe RSV bronchiolitis in pre-term infants. A further major limitation is the lack of long-term follow-up in the studies included. This meant that our outcome used was recurrent wheezing rather than true asthma. Further studies with longer follow-up are required to address the question of RSV prevention and asthma causation.

The main strength of this systematic review and meta-analysis is the thorough literature search, careful study selection with strict inclusion criteria, and comprehensive assessment of bias using the established GRADE approach. Furthermore, the findings of this study are in support of existing evidence for the association between RSV bronchiolitis and recurrent wheeze/asthma.

## Conclusion

It is still unclear whether administering RSV prophylaxis to pre-term infants reduces the risk of respiratory complications later in childhood. On the basis of pre-specified sub-group analyses excluding low-quality studies, and focusing on late pre-term infants, we conclude that potential long-term benefits cannot be ruled out. This review highlights the need for longer-term high-quality clinical trials investigating RSV prophylaxis on the outcome of asthma, as current evidence is of very limited quality. In particular, there should be a focus on these benefits in late pre-term infants (32–36 weeks).

## Supplementary Information

**Additional file 1.** PRISMA 2009 Checklist.DOCX 19 kb)

**Additional file 2.** Studies excluded based on full text, with full citation and reasoning.

## Data Availability

All data was obtained from studies included in reference list [32–39]

## Abbreviations

GRADE: Grading of Recommendations Assessment, Development and Evaluation
RSV: Respiratory syncytial virus
