# Supplementary material for: Respiratory syncytial virus prophylaxis for prevention of recurrent childhood wheeze and asthma: a systematic review
Source: Syst Rev. 2020 Nov 25;9:269. doi: 10.1186/s13643-020-01527-y (PMC7690183; doi:10.1186/s13643-020-01527-y)
Supplement: Supplementary file 2 — Additional file 2. Studies excluded based on full text, with full citation and reasoning. [file 13643_2020_1527_MOESM2_ESM.docx]

**Additional file 2.** Studies excluded based on full text, with full citation and reasoning.

| **Study Excluded by Full Text** | **Reasoning** |
| --- | --- |
| Simoes EAF, Carbonell-Estrany X, Rieger CHL, Mitchell I, Fredrick L, Groothuis JR, et al. The effect of respiratory syncytial virus on subsequent recurrent wheezing in atopic and nonatopic children. Stephan V  Berner R, Carbonell-Estrany X, Figueras J, Pedraz C, Remesal Escalero A, Fraga J, Martinez Soto MI, Perez Frias J, Blasco Alonso J, Narbona E, Maldonado Lozano J, Roques V, Salas Hernandez S, Tabeada Perianes M, Fernandez Trisac JL, Echaniz I, MG, editor. J Allergy Clin Immunol [Internet]. 2010;126(2):256–62. Available from: <http://ovidsp.ovid.com/ovidweb.cgi?T=JS&PAGE=reference&D=emed11&NEWS=N&AN=50985266> | *This study was carried out by the same researchers in the same cohort of infants using the same data as the study titled ‘Palivizumab prophylaxis, respiratory syncytial virus and subsequent recurrent wheezing’, Simoes EAF et al 2007. This time they were comparing the protective effects of Palivizumab in infants with and without a family history of atopy, therefore this does not strictly comply with our inclusion criteria, and any possible data obtained about rates of recurrent wheeze with monoclonal antibody prophylaxis would be the same data as the previous study.* |
| Yoshihara S, Kusuda S, Mochizuki H, Okada K, Nishima S, Simoes EAF, et al. Effect of palivizumab prophylaxis on subsequent recurrent wheezing in preterm infants. Adachi Y  Inoue T, Sakamoto T, Tsutsumi H, Fujisawa T, Hosoya M IM, editor. Pediatrics [Internet]. 2013;132(5):811–8. Available from: <http://ovidsp.ovid.com/ovidweb.cgi?T=JS&PAGE=reference&D=med7&NEWS=N&AN=24127479> | *This study is an earlier version of the study titled ‘Palivizumab Prophylaxis in Pre-term Infants and Subsequent Recurrent Wheezing. Six-Year follow-up Study.’ (Mochizuki et al 2017). This study did meet our inclusion criteria however we made the decision to use the version with the longer follow-up period.* |
| M.O. B, M.M. R, J.M. M, P.L. W-S, A. M, J.L.L. K. Respiratory syncytial virus and recurrent wheeze in healthy preterm infants. N Engl J Med [Internet]. 2013;368(19):1791–9. Available from: http://www.nejm.org/doi/pdf/10.1056/NEJMoa1211917 | *This was the MAKI trial at an earlier stage of follow-up (1 year). This paper met our inclusion criteria however we decided to include the later version of this trial where the infants were followed up after 6 years.* |
